# COVID-19-related arterio-ureteral fistula formation in a post-transplant patient

**DOI:** 10.1016/j.radcr.2022.12.015

**Published:** 2023-01-05

**Authors:** Adam L. Richardson, Olivia K. Richardson, Nikolas J. Touloumes, Nana Y. Ohene Baah

**Affiliations:** a Department of Radiology, University of Louisville School of Medicine, 530 S Jackson St, Louisville, KY 40202, USA; b Department of Surgery, University of Louisville School of Medicine, 530 S Jackson St, Louisville, KY 40202, USA; c Department of Medicine, University of Louisville School of Medicine, 530 S Jackson St, Louisville, KY 40202, USA

**Keywords:** Arterio-ureteral fistula, COVID-19, Renal transplant, Pancreatic transplant, Interventional radiology

## Abstract

Arterio-ureteral fistulas (AUF) are extremely rare and are not commonly suspected in the setting of patients with post-renal allograft transplantation. The diagnosis, while uncommon, can be potentially lethal which is only exacerbated by the clinical conundrum associated with their under-recognition and various treatment algorithms. This case identifies a unique patient with a history of 2 failed renal transplants who presents with new onset intermittent hematuria after contracting coronavirus disease 2019 (COVID-19). Despite the patient having his second renal transplant graft embolized, the patient continued to present with refractory hematuria, leading to the realization and identification of an AUF to his right renal graft. This sequence of events brings forth a case of unique significance, underscoring the potential ramifications that COVID-19 poses to the renal transplant population.

## Introduction

An arterio-ureteral fistulas (AUF) is a disseminating bridge between an arterial structure and a ureter. AUFs are caused by factors that result in acute or chronic inflammation at the surrounding sites involving an artery and ureter. Common risk factors include recent surgery, cancer, radiation, and even more commonly, chronic ureteral stent placement. The main complaint on presentation is intermittent or massive gross hematuria, with the majority presenting with the later. Accompanying symptoms may consist of hemorrhagic shock, abdominal or flank pain, and infection. A 2022 review of AUFs was only able to identify 445 patients from the years 1908 to 2020, indicating this is an exceedingly rare diagnosis, as only 1 patient was identified to have acquired an AUF that fell outside of the acute surgical transplantation period. When performing a literature review at the time of this case report, there are no reported cases of this nature to involve or describe the individual effects of coronavirus disease 2019 (COVID-19) on a dual renal and single pancreatic transplant recipient who sustained separate pathologies of graft intolerance syndrome and an AUF [1].

This report will discuss a unique case emphasizing a potential singular effect of COVID-19 in a post-renal transplant patient while bringing awareness to the added benefits of including interventional radiology in multidisciplinary teams.

## Case report

A 21-year-old male with a past medical history of diabetes mellitus type-1 with 2 prior renal transplants and pancreatic transplant presented to the emergency department with a complaint of extreme fatigue and hematuria after recently testing positive for COVID-19. Of note, he was COVID-19 vaccine-naïve at the time of contracting the virus. Additionally, he started to notice blood in his urine 5 days after his positive COVID test, worsening until his eventual arrival at the emergency department.

A pertinent backstory regarding the patient history reveals that at the age of 6 years old, the patient suffered from E. coli-related hemolytic uremic syndrome, leading to his eventual end-stage renal failure and his later living donor renal transplant. The kidney was placed in his right iliac fossa and anastomosed to the distal aorta and inferior vena cava. The allografted kidney maintained viability for thirteen years, after which point it failed and the patient was transitioned to hemodialysis. The following year, the patient received a pancreatic and additional renal transplant, this time from a deceased donor. The kidney was placed in the left iliac fossa and the pancreas was placed in the right iliac fossa at the time of surgery. The pancreas was attached with a Y-graft end-to-side anastomosed at the right common iliac artery, as the pancreatic head remained cephalad. The left renal allograft failed after one-year following transplantation and he was placed back on hemodialysis. It was less than 1 month later that he contracted COVID-19 and presented to our emergency department.

At the emergency department, the patient's hemoglobin level was 4.6 g/dL on admission with symptoms concerning for hypovolemic shock. He was subsequently admitted to the intensive care unit for acute monitoring and treatment and placed on renal replacement therapy. Even after medical therapy and multiple transfusions, the patient continued to remain anemic with persistent intermittent hematuria and hypertension. The nephrology team suspected the possibility of graft intolerance syndrome during their initial assessment, which seemed more apparent as time progressed. On day 4 of his hospital admission, the patient had an episode of severe abdominal pain with another acute drop in hemoglobin from 7.6 to 5.8, triggering a computed tomography (CT) scan of the abdomen and pelvis. Imaging revealed that the patient had abnormal high-density fluid within the pelvis, concerning for blood products, with a diffusely edematous left renal transplant and no visible evidence of hydronephrosis or hydroureter. Based on the clinical history and presentation, in addition to physical exam findings and imaging results, the patient was diagnosed with graft intolerance syndrome. Steps were then taken to correct the concerning issue of graft intolerance syndrome with arterial embolization of the transplanted left kidney. A day later, the patient was taken to the interventional radiology (IR) suite for this procedure. An angiogram of the left transplant kidney during the procedure showed stenosis of a segmental renal artery with easy reflux into the iliac artery and no early filling venous abnormalities. Post-embolization angiogram demonstrated coil embolization of the transplanted kidney from the subsegmental renal arteries to the main renal artery. The patient's hemoglobin subsequently appeared to stabilize, and he was discharged. His hemoglobin remained within 9-10 g/dL following the procedure.

Following discharge, the patient would later frequent the emergency department on 2 different occasions for recurrent complaints of abdominal pain with no identifiable cause other than the first visit where he was admitted for diabetic ketoacidosis resulting from sepsis secondary to pneumonia. A CT scan performed during the second occurrence identified new mild hydronephrosis of the right kidney; however, this was never investigated further due to prior graft failure.

**Fig. 1:**
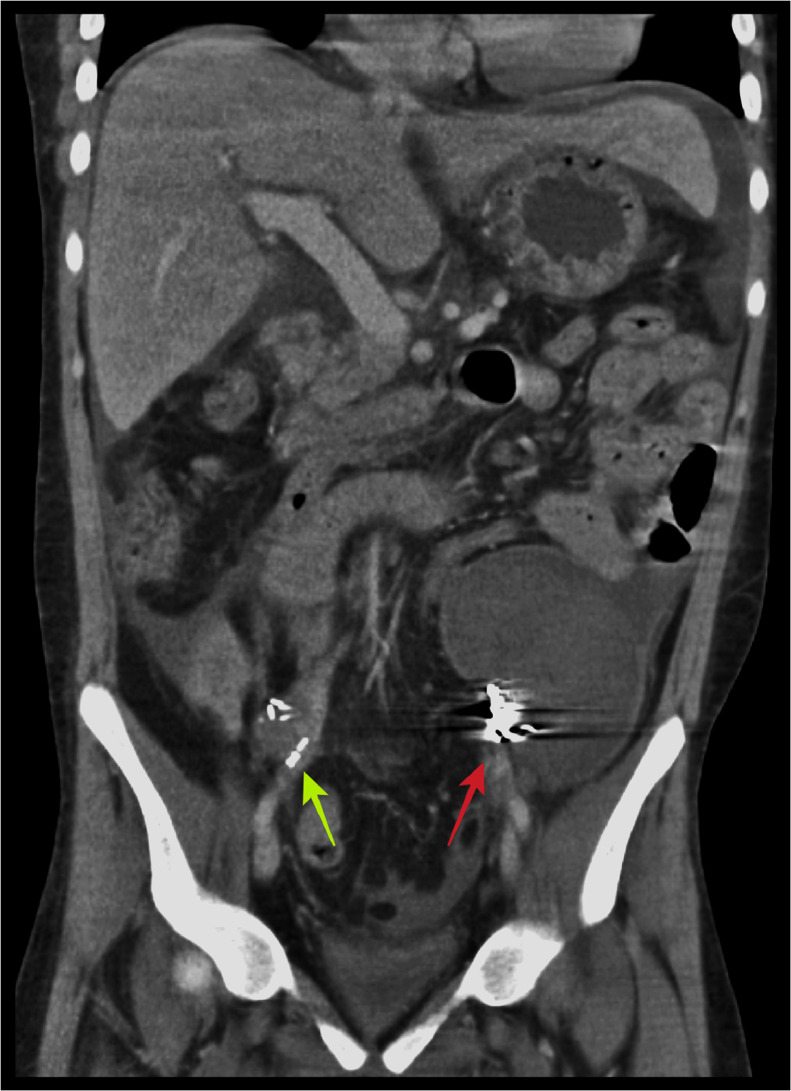
Coronal CT imaging of the patient's abdomen and pelvis following embolization of the left renal allograft. Red arrow highlights the embolization coils from the left renal graft embolization. The left renal graft remains swollen, edematous, and without enhancement except for a slight degree of enhancement at the cortical rim, due to proximal perforators supplying the cortex. The overall graft has structural distortion from necrosis. Green arrow identifies the surgical clips from the pancreatic implant, overlying the right iliac artery.

Two months after the initial left renal transplant embolization had passed, the patient resurfaced again at the emergency department but this time with another episode of gross hematuria. Urology was consulted and cystoscopy identified gross hematuria emitting from the right transplant ureteral orifice with the orifice too stenotic for scope advancement leading to a 6 French stent being placed. Following stent placement, the patient was initiated on continuous bladder irrigation therapy due to the marked clot burden within the bladder. At a multidisciplinary conference, it was decided that the failed right transplanted renal graft would now require embolization (Fig. 1).

**Fig. 2:**
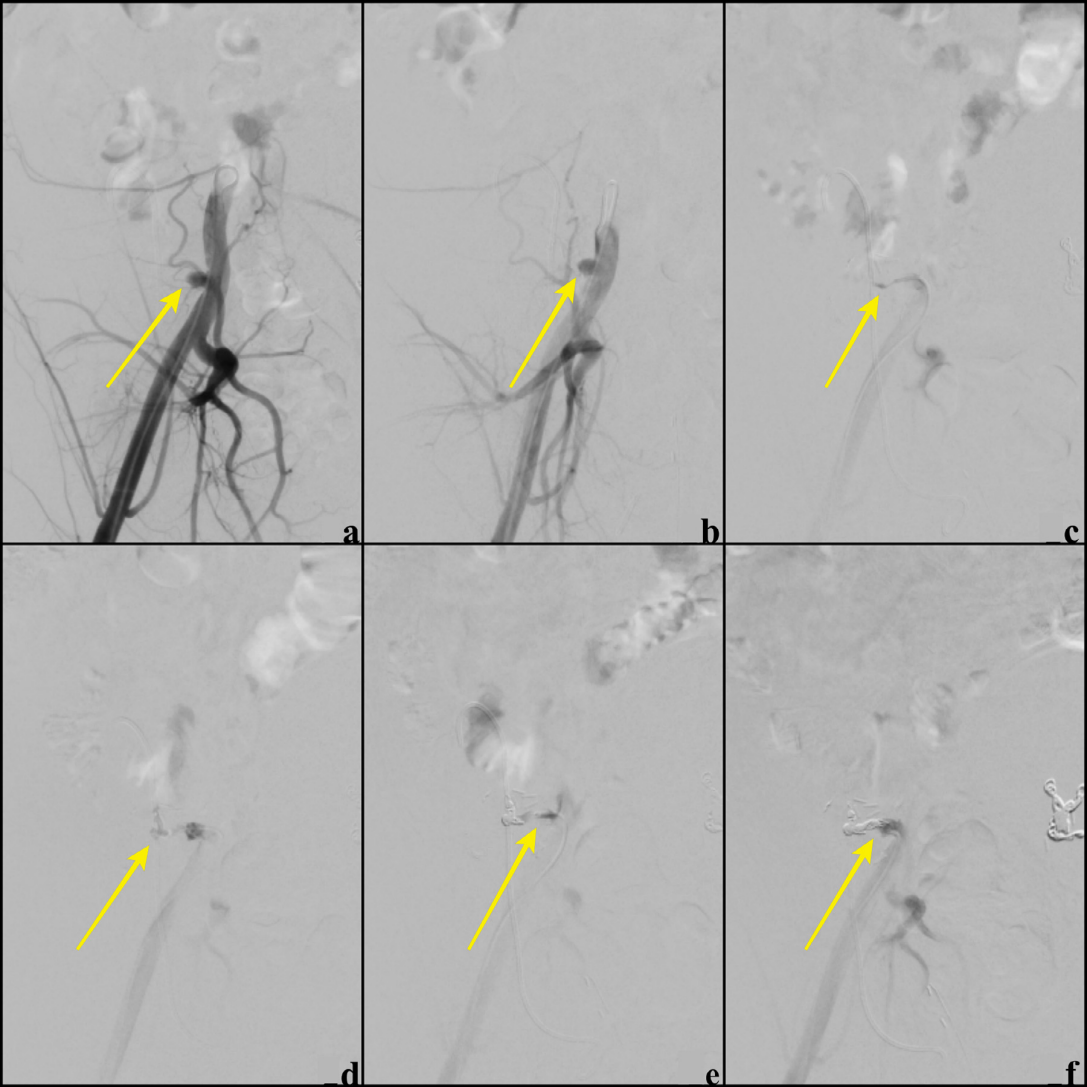
Arteriogram of the right external iliac with selective catheterization and embolization of the AUF between the grafted right pancreatic artery and ureter. (a) The initial run of the right iliac arteriogram. (yellow arrow) pointing to the truncated pancreatic artery. (b) Yellow arrow demonstrates the persistence of the truncation with an additional run in closer proximity to the pancreatic artery after catheter retraction. (c) Selective catheterization was performed. Yellow arrow points out the narrow tract that moves towards the ureteral stent, correlating to the right ureter. (d) After additional contrast delivery, the contrast migrates cephalad to further outline the fistulous communication with the ureter. Yellow arrow points toward the distal area of migration. (e) Embolization coils are deployed distally within the tract. Yellow arrow shows contrast halting proximal to the tract. (f) Additional coils were placed proximally after distal coils were appropriately placed. Yellow arrow highlights the embolized tract, representing the prior communication between the pancreatic artery and the right ureter.

**Fig. 3:**
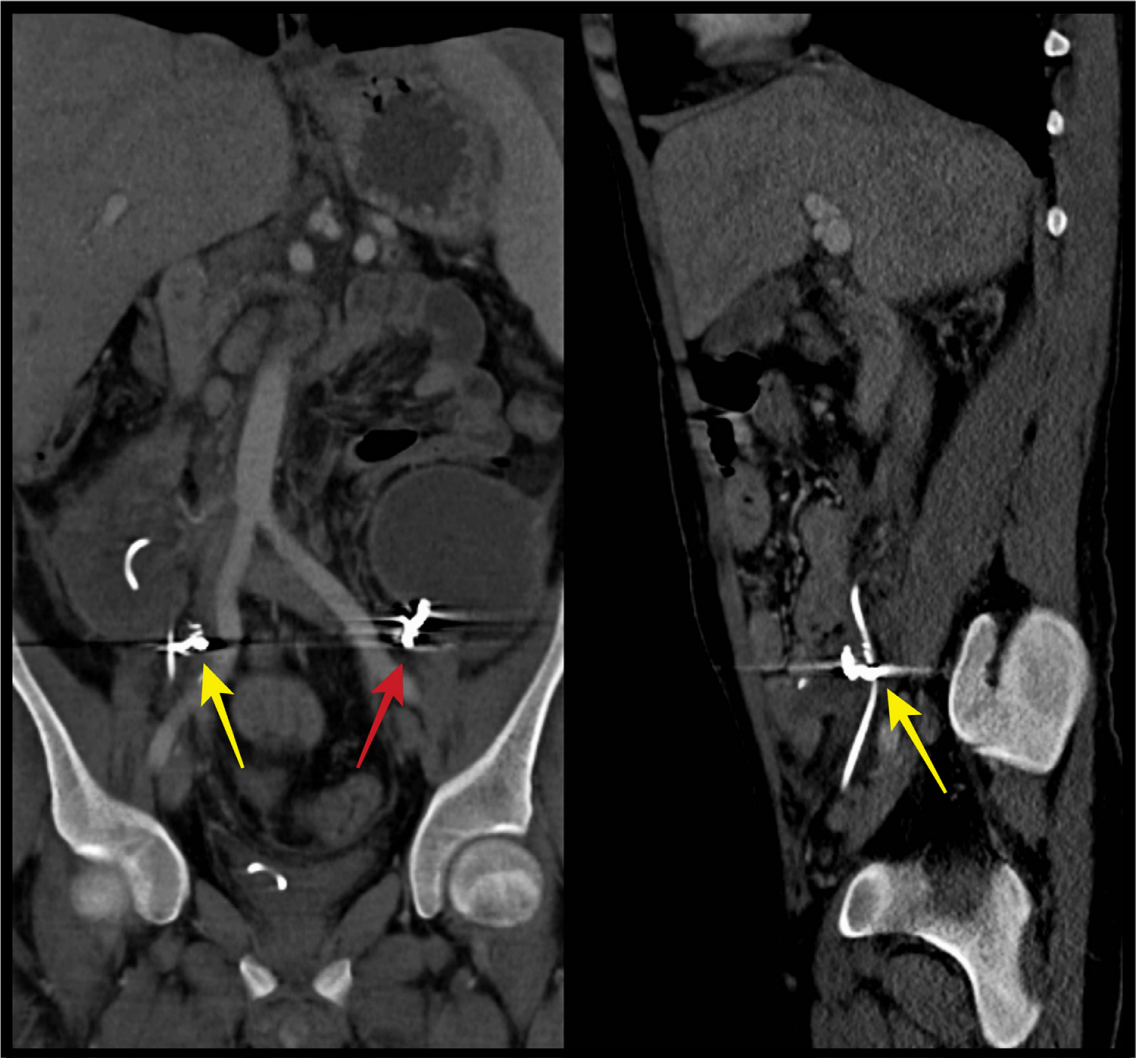
Coronal and Sagittal CT imaging of the patient's abdomen and pelvis following AUF embolization. Red arrow redemonstrates the embolization coils from the preceding operative embolization of the right renal transplant that were previously identified in Figure 1. Yellow arrows identify the newly deposited embolization coils within the coronal and sagittal images. The coils were implanted within the fistulous tract between the pancreatic transplanted artery and the right ureter.

On the day of the procedure, the patient's hemoglobin dropped from 9.6 to 8.7 g/dL. The patient was taken to the IR suite for a right transplant renal angiogram with embolization. Before embolization, diagnostic runs were performed. This is what lead to the identification of the abnormalities at the right pancreatic arterial graft, and a non-selective angiogram of the right pancreatic artery was performed to further investigate the finding of what would later be identified as a fistulous communication between the pancreatic artery and the right ureter. In a coaxial fashion, a microcatheter was advanced through the communicating tract, and coil embolization was performed with 14 mm x 15 cm and 16 mm x 20 cm coils. Post embolization angiogram was performed, demonstrating satisfactory resolution of the AUF between the pancreatic artery and the ureter. Hemoglobin remained stable following the procedure and the ureteral stent was removed. Figs. 2 and 3 demonstrate the embolization coils and the anatomic relationship that they share with the pancreatic allograft.

## Discussion

The primary concern for all transplant patients is graft failure. This is no different for renal transplantation. Renal graft failure has a failure rate of about 10% at 1-year and 40% at 5-year intervals when accounting for both living and non-living donors. The numbers have improved over the years due to modern medical advances in immunosuppressive therapies and improved surgical techniques [2]. However, even in the best scenario, most allografts will statistically fall short and fail before the recipients' expiry. When the graft does fail, there is not yet an established consensus on standardization for how to manage a non-functioning renal graft. One thing that is known is that if a graft is retained without embolization following graft failure, they are at risk of developing graft intolerance syndrome. The nature of graft intolerance syndrome revolves around the immunologic intolerance of a failed allografted renal transplant(s) left in situ and is associated with a similar and non-distinct set of signs and symptoms, not limited to but including fever, localized pain, malaise, hypertension, refractory anemia, and hematuria. Imaging can also assist in confirming the diagnosis, as the kidney will characteristically appear enlarged and without evidence suggestive of infection or malignancy. Ruling out infection is pivotal for all transplant patients and can be arduous when considering the sheer number of bacterial, fungal, and viral infections that can exploit the immunocompromised state of these patients. After a diagnosis of exclusion is obtained, the standard of care is that a patient is offered a transplant nephrectomy or renal vascular embolization. Graft intolerance syndrome is the main indication for transplant nephrectomy or embolization after post-renal graft failure, with embolization proving to be a superior option relating to outcomes of morbidity and mortality [3,4]. Regardless, an in-depth conversation should be had with the patient about their options and both procedures.

It is common practice that if an allografted kidney has been in place for more than 1-2 years, it can be left in situ, and this is why our patient had retention of both his failed renal transplants. The patient also remained on immunosuppressive therapy even after the failure of both renal transplants, due to the remaining viability of his pancreatic transplant and for the benefit of preserving any remaining residual kidney function and circumventing graft intolerance syndrome by minimizing allosensitization [5].

COVID-19 causes multi-system dysregulation through profound inflammation. This commonly results in symptoms associated with patterns of vasculitides and the overall threat to vascular structure and function, acting through impairment of the immune system, renin-angiotensin-aldosterone system (RAAS), and thrombotic system, leading to widespread systemic effects. The acute onset of COVID-19 initiates this assault, but this inflammation when associated with an altogether impaired immune response causes mild chronic inflammation even after the acute phase, altering the structural properties of arterial anatomy well within the post-acute phase; lasting up to 4 months. During this post-acute period, the inflammatory effects of COVID-19 alter the arterial components similar to other chronic inflammatory diseases, by a means of inflammation that results in arterial stiffening and later adverse cardiovascular events [6]. Indicatively, these turmoiled effects are identified within the vascular network of a native kidney and transplanted grafts. In a study of 147 non-transplant patients, up to nearly half of the patients exhibited hematuria during their hospitalization, highlighting the comparative nature that COVID-19 plays on the nontransplant community.

One particular study investigating the effects of COVID-19 on transplant recipients, consisted of 475 patients, 195 of whom were hospitalized, 69 were admitted to the ICU, and 47 died after a median length of 15 days, emphasizing the high risk of severe illness once renal transplant patients acquire COVID-19 [7]. After reviewing the devastating sequelae COVID-19 enacts on renal transplant patients, it is a direct and realistic notion that this is a possible inciting factor, with inflammatory disruption of the allografted kidney causing graft intolerance syndrome and also leading to the formation of an AUF [8]. All other evidence to suggest an alternative cause was negative; infectious panels, evidence for malignancy, and low-dose immunosuppressive therapy were all negated. The coincidental nature of the previously described events occurring within 60 days of contracting COVID-19 allows for this conclusion to be drawn.

## Conclusion

The correlative relationships brought forth in this case report regarding COVID-19 and the formation of AUFs are supportive but limited to postulation. Specifically, a higher sample size is required before cases of this nature can imply anything past that of a simple correlation. This is expected, given the only recent emergence of COVID-19 within the last 4 years and the rarity of this patient's presentation. A limitation, while beneficial due to the improved complication rates associated with renal embolization versus transplant nephrectomy, is that pathologic specimens were not procured during this case, limiting further substantiation and microscopic review of the allografts. The realistic hope is that this case will not only provide a framework for future studies but will assist others with the treatment and management of this select population, especially as the utilization and requirement of transplantation are only expected to increase with the further advancement of medical technology and the increasing longevity of an aging population.

## Patient consent

The patient has provided his consent, confirming that written, informed consent has been granted for the publication of their case.

## Appendix Supplementary materials

Image, application 1
