# Supplementary material for: COVID-19-related arterio-ureteral fistula formation in a post-transplant patient
Source: Radiol Case Rep. 2023 Jan 5;18(3):1015–20. doi: 10.1016/j.radcr.2022.12.015 (PMC9815792; doi:10.1016/j.radcr.2022.12.015)
Supplement: Supplementary file 1 [file mmc1.docx]

**COVID-19 related arterio-ureteral fistula formation in a post-transplant patient**

Adam L. Richardson DO1, Olivia K. Richardson MD, PharmD2, Nikolas J. Touloumes DO3, Nana Y. Ohene Baah MD1

1Department of Radiology, University of Louisville School of Medicine, 530 S Jackson St, Louisville, KY 40202, USA

2Department of Surgery, University of Louisville School of Medicine, 530 S Jackson St, Louisville, KY 40202, USA

3Department of Medicine, University of Louisville School of Medicine, 530 S Jackson St, Louisville, KY 40202, USA

**Running Title:** COVID-19 related arterio-ureteral fistula formation in a post-transplant patient

**Word Count:** 2,369
